# Design, Modeling and Implementation of Digital Twins

**DOI:** 10.3390/s22145396

**Published:** 2022-07-20

**Authors:** Mariana Segovia, Joaquin Garcia-Alfaro

**Affiliations:** Télécom SudParis, SAMOVAR, Institut Polytechnique de Paris, 19 Place Marguerite Perey, 91120 Palaiseau, France; segovia@telecom-sudparis.eu

**Keywords:** digital twin, digital model, control system, cyber-physical system, network simulation, software simulation, system simulation, Industry 4.0

## Abstract

A Digital Twin (DT) is a set of computer-generated models that map a physical object into a virtual space. Both physical and virtual elements exchange information to monitor, simulate, predict, diagnose and control the state and behavior of the physical object within the virtual space. DTs supply a system with information and operating status, providing capabilities to create new business models. In this paper, we focus on the construction of DTs. More specifically, we focus on determining (methodologically) how to design, create and connect physical objects with their virtual counterpart. We explore the problem into several phases: from functional requirement selection and architecture planning to integration and verification of the final (digital) models. We address as well how physical components exchange real-time information with DTs, as well as experimental platforms to build DTs (including protocols and standards). We conclude with a discussion and open challenges.

## 1. Introduction

A Digital Twin (DT) is composed of computer-generated models representing physical objects. Such models continually adapt to operational changes based on data collected directly from the physical objects. DTs can be implemented over cyber-physical systems. Examples range from isolated robots to complete environments, such as smart cities and 5G networks [1].

DTs are expected to forecast the state evolution of the physical object using the exchanged data. Such exchange from physical to the virtual domains can be conducted following real-time constraints. DTs can also supply a system with information and operating status, providing capabilities to create new business models. It is also possible to make more accurate predictions and provide situational awareness. Other uses of DTs include reducing costs and risks and improving efficiency, security and resilience.

Incorporating a DT presents many advantages for the management of the physical objects. For example, to predict and optimize the system behavior, i.e., using models or even machine learning techniques, it is possible to predict future behaviors of the system and improve the process productivity. A common use of DTs for this purpose is to prevent service disruption under maintenance situations. A DT can also be used to perform continuous monitoring via real-time data acquisition. This allows the DT to provide information to make better business decisions and control the physical system. DTs can provide a testing platform to verify different scenarios to choose the most efficient one and to increase the system performance. Another common use of DTs is to improve security and resilience, due to DT’s ability to detect malicious actions on a system. In addition, it allows for a better risk assessment to test various *what-if* cases that may affect the physical objects.

Academic research and industrial interest on DTs has grown in the last few years, especially due to its future potential and wide range of useful applications. Gartner has listed DTs as one of the top ten technology trends for years to come [2], and it was classified as one of the technology pillars of Industry 4.0 in [3]. Building a DT is still a complex process; it requires many steps of design, modeling and implementation to obtain a functional system. Nevertheless, DT modeling is still a challenge [4]. To start, there is a lack of standards heading the construction of DTs, as well as a lack of mature methodologies to lead the process from the initial design to the simulation of the complete system. Even though multiple approaches and technologies have been proposed, it is necessary to have a design procedure which can support the selection of techniques and tools to build a DT based on the system requirements.

Our work aims at answering those aforementioned issues. We propose a concrete process to design DTs, as well as creating models and using existing tools to implement them. Our proposal comes from a systematic review of the existing literature’s building techniques for DTs. We provide in-depth insights regarding the design, modeling and development techniques, i.e., how we can build a DT starting with a Physical Object (PO) that we want to model and reproduce by software. We formulate the following research questions: how can we model a DT? How can we design a DT architecture? how can we synchronize the data in real-time between both components, and what are the main challenges in developing DTs? We also cover critical challenges and evolution trends.

**Figure 1:**
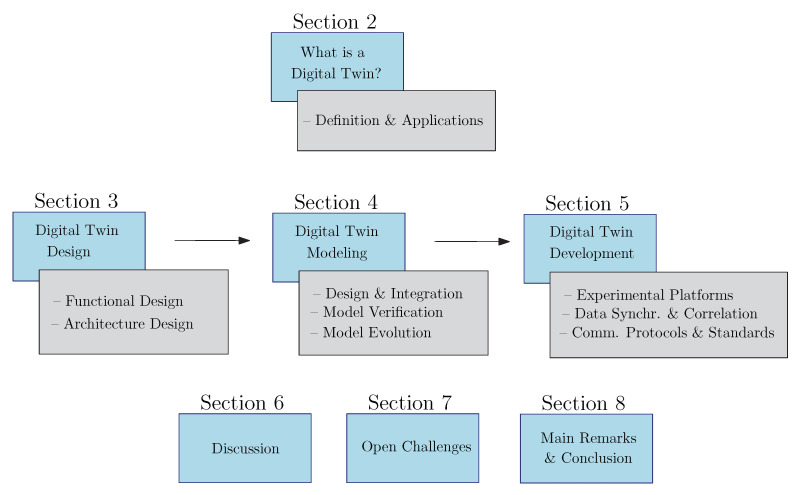
Organization of our work.

To conduct our survey, we followed a systematic literature review methodology [5], based on the following terms:

- **Collection Strategy**—We used a keyword search to make the first selection of potentially relevant scientific publications published in the last five years. We considered databases such as Google Scholar, IEEE Xplore, DBLP and Science Direct to collect the publications. Articles were filtered out with the keyword *digital twins*. Duplicate articles were deleted and the most relevant works were filtered according to their titles and abstracts. We included other publications using references from the first dataset. This second dataset may contain publications older than five years if the content was deemed relevant for the survey;
- **Dataset Filtering**—The inclusion criteria for our study was based on the following conditions: (1) the DT should be approached from a computer system viewpoint; and (2) the proposal should be useful for the simulation and optimization of the real system. The exclusion criteria was based on the following conditions: (1) no scientific articles written in a language other than English or with full content access denied; and (2) the fundamental concepts, their related properties or the DT implementation were not adequately described;
- **Literature Classification**—We aimed at clarifying how to build DTs, i.e., which process to follow to design the different parts of the platform and which existing tools can be used. Hence, the selected literature was analyzed and classified based on our proposed design procedure to design, model and develop a DT. We organize the paper using the taxonomy shown in Figure 1. We propose a three-step procedure that corresponds to the chronological order of the activities required to implement a DT. The first step explains how to design a DT, i.e., how to define the functional requirements and the system architecture. The second step explains how to model a DT. This step requires creating system models that represent different aspects of the PO and integrating them. Finally, the third step explains how to implement a DT, i.e., which are the existing platforms, frameworks and tools for developing a DT, how to synchronize the data between physical and virtual objects and the existing communication protocols and standards for the information exchange.

**Paper Organization: **Section 2 provides general background information on DTs. Section 3, Section 4 and Section 5 provide, respectively, our findings on designing, modeling and implementing DTs. Section 6 and Section 7 provide a discussion, the research challenges and the future perspectives of the work. Section 8 concludes the paper.

## 2. What Is a Digital Twin?

The DT concept was fist introduced in 2002 by Michael Grieves, and since then, the concepts and its associated technologies have evolved significantly. A detailed review of the DT history can be found in [6,7].

A DT is composed of a real space containing a physical object, a virtual space containing a virtual object and a link for data flow from the real space to the virtual space and information flow from the virtual space to the real space [8]. Hence, a DT can be seen as a digital copy of a physical object (PO) and its process [9]. The PO’s behavior is converted into a virtual object (VO); both components are interconnected with a high level of synchronization between them [10,11]. This link enables data exchange allowing physical–digital convergence [12]. DTs are normally used in Cyber–Physical Systems (CPS) and networks as they integrate simulation of a physical product. For that, DT models the mechanical, electrical, software and other properties to optimize the physical product based on the updated real-time data synchronized from sensors [13].

A DT may integrate different physical laws, as well as different scales or probabilities reflecting the underlying state of the PO. This can be based on, e.g., historical data of the physical model [14,15]. Different physics models cooperate with different system descriptions, such as aerodynamics, fluid dynamics, electromagnetics and tensions. Moreover, the use of different scales adapt the simulation to the required depth in terms of temporal constraints. For instance, users can navigate different parts of the DT, visiting different elements of each component. This can be complemented with the adoption of different probabilistic models associated with each building block, e.g., to augment the prediction capabilities of the DT.

One characteristic property of DTs is the connection and real-time data exchange allowing continuous or periodic synchronization of the Virtual Object (VO) and PO [7]. The direction of the information is mainly from the PO to the VO. However, the VO may send data and information to the PO [16]. The VO processes the historical data, such as maintenance history and operational history from the PO along with real-time dynamic data sensed and collected from multiple sources describing the PO status and its surrounding environment status. The VO sends information, for example, to correct some states or errors, reinitialize a machine after a break or synchronize states with other cooperating robots. It may produce additional predictions to enhance system maintenance [7].

Some authors [17,18,19] propose the notion of a *Cognitive DT* which is inspired by advances in cognitive science. They propose to add the following functions to the DT: selective attention or focus; perception by forming useful precepts from raw sensory data; memory based on encoding and retrieving knowledge; reasoning by drawing inferences from observations, beliefs and models; learning from experiences, observations and teachers; and problem-solving to achieve goals. Mortlock et al. [19] propose using graph learning as the pathway towards creating cognitive functionalities in DTs. The graph is built using data models that use a Graph Neural Network (GNN).

As stated in the literature, DTs are a development of modeling and simulation technology. However, they are also different, as they break through the limitations of these techniques by integrating Internet of Things (IoT) technologies. Next, we outline the main differences between these concepts.

### 2.1. Digital Models vs. Digital Shadows vs. Digital Twins (DT)

The main difference between DTs, digital shadows and digital models is the nature and direction of the data flow between the physical and virtual systems.

A digital model is a digital version of an existing or planned physical object and there is no automatic data exchange between the physical model and the digital model. This means that once the digital model is created, a change made to the physical object has no impact on the digital model either way [4].

In the digital shadow scenario, there is data flowing from the physical object to the digital model. Hence, the model is actualized with new information from the real world.

In a DT, the data flows are between the existing physical object and the digital object, and they are fully integrated in both directions. A change made to the physical object automatically leads to a change in the digital object and vice versa. In DTs, the flow of data is automatic and synchronizes the digital object with the current status of the physical one, also sending control information to it.

The fundamental element of a DT is the connection between digital and physical systems carrying data and control information between them. Having this data, a DT can supply every required piece of information about the physical system in a real-time manner, which constitutes the optimal target for DTs.

### 2.2. Digital Twins vs. Simulations

The major difference between a DT and a simulation is the data interconnection that allows exhanging information between the PO and the VO, i.e., a simulation predicts future states of a physical system based on a set of initial assumptions [20]. However, a DT tracks the current and past states of the PO that is being used in operation and is being simulated within the VO. Traditional simulation methods are of limited capability in evaluating system performance and considering the physical part of the system [13].

Often, the computational models used to infer the current state of the PO are the same models which can be used in simulation to predict future states. The simulation models can provide additional decision-making information for optimizing future operations, forecasting degradation mechanisms and predicting future failures.

**Figure 2:**
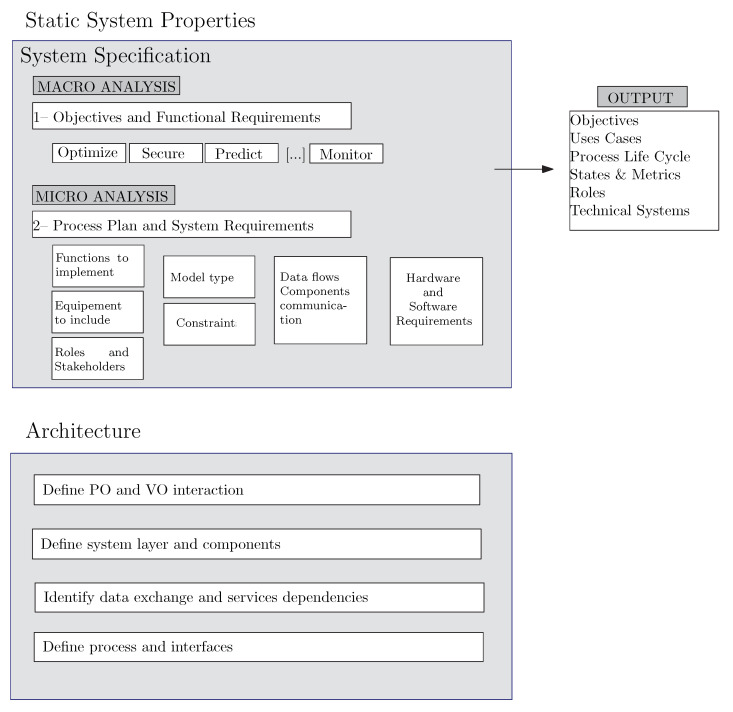
Methodological design of Digital Twins.

Considering this DT definition, we analyze how to build a DT. The whole process is shown in Figure 2. In the next Section, we explain the design phase, including how to create a functional design and the system architecture.

## 3. Digital Twin Design

The first step of the process to build a DT requires modeling the static properties of the system. The objective is to determine the system requirements and constraints, including the functions and functional decomposition. Model data flows and communication should also be considered, as should the architecture and logical structure of the system and the technical requirements to implement the solution, including hardware and software parts.

The VO is usually designed as a mirror of the PO. Thus, both components may co-exist during the DT’s life cycle. In case the PO already exists at design time, the early phase will then focus on connection tasks from the VO to the PO. It can also happen that the PO component does not exist at all during the design phase of the VO. In such a case, a prototype PO can be made in order to lead the construction of the VO, i.e., the prototype and the VO are combined in order to validate some design choices. Regardless of the existence of the PO at design time, the VO shall follow and mirror either the PO or its prototype in the end. Simulation can be used to predict some malfunctioning if the product is stressed or used in critical situations [16].

An important characteristic of the DT is its continuous synchronization with the production system and its evolution; for example, changes in wiring or physical or fixation position should be also considered in the VO. For this reason, during the system design, the system must be designed to be adaptable and consider updated engineering models or processes modifications.

### 3.1. System Specification

The function model determines, firstly, the objective and functional requirements of the DT, i.e., if the DT will help optimize, secure, monitor or predict the physical process. Then, it is necessary to analyze which functions or activities of the physical process will be included in the DT. For that, we need to elaborate a *process plan* considering both the physical process and the DT functionalities, determine which equipment will be included, which modeling aspect will be included (for example, mechanics, 3D space, electronics, or others) and the relationship among both components, i.e., how both components are going to exchange information. Moreover, we have to determine the non-functional DT requirements [21].

#### 3.1.1. Functional Requirements

This phase defines the objective and functional requirements of a DT to create its specification. It also defines which information we need to extract and describe based on the PO. The objective is to define accurate DT requirements and to obtain as much information as possible to build the system. The successful analysis of the requirements is key for the system modeling. In the literature, DTs have been used for different purposes, such as optimizing, securing, monitoring, predicting, training or improving the physical process. Normally, a DT combines one or more of these functionalities. Table 1 summarizes some examples.

*Optimization*—All the data generated by the DT can be analyzed with advanced data techniques to provide precise information. As a result, a DT can improve the performance of a system, improve its efficiency, reduce costs or risks and improve decision making. In this case, it is needed to define specific and measurable system objectives, as well as cost functions, to control the system and evaluate them. Some research proposals that build DTs to optimize a process are cited as follows.

Stan et al. [22] propose a system that uses multiple robot cells for product-on-pallet distribution. The DT helps in the distribution planning, activity scheduling and resource allocation, resource health monitoring, robust process control and maintenance of resources. It optimizes the activities in three process stages: palletizing, storage and shipment of products. Wang et al. [23] propose a 5G DT for cost-efficient and performance-optimal management of the network. The approach creates a virtual representation of slicing to simulate its behaviors and predict and optimize the time-varying performance. The approach uses a Graph Neural Network model to learn the insights directly from slicing-enabled networks. Bhatti et al. [24] analyze how DTs can be implemented in electric cars to increase energy efficiency and reduce greenhouse gas emissions. An et al. [25] proposed a DT to reduce methane emissions that cause global warming. The framework uses drones to measure real-time data about the emissions. Bottani et al. [26] propose an optimized DT to optimize and prevent high-risk events for a beverage pasteurization system. It uses virtual modeling of the process based on machine learning techniques. Guo et al. [27] built a DT that optimizes, reduces complexity and reduces uncertainty in the layout of assembly positions in the manufacturing industry, where the product remains at one assembly point and workers, equipment and materials are moved around according to the assembly plan. The DT considers customer demands, production capacity constraints and real-time ticket pool management mechanisms to manage production activities optimally. It helps to make production decisions and helps operators efficiently complete their tasks with error-free operations. Gonzalez et al. [28] present a DT of a transportation system to evaluate its condition and potential corrective solutions. The authors use the physics model to guide the system, detect faults, improve energy efficiency and test what-if cases.

*Security Improvement*—Another application of DTs is to improve the risk assessment, detection and evaluation of countermeasures to protect a system. For example, DTs can run in parallel to a CPS to analyze the security and safety of the system during its operation. Eckhart et al. [51] survey the application of a DT for a cybersecurity application in CPS. CPSs have two interdependent layers, the physical layer and the cyber layer. Both need to be secured to protect the system operation from threats. The main uses are to improve the security design, to detect misconfiguration, pentest, compliance improvement and security training. In addition, they allow for analyzing detection, mitigation and resilience techniques in the VO before deploying the solution on the physical controlling components. The information in the DT allows for detecting attacks and also restoring the system state using data from the VO. Another benefit is that the DT may be used to elaborate a what-if analysis, resulting in a better risk assessment. For example, it is possible to perturb the system to test unexpected scenarios and analyze the response of the system.

Some proposals that use DTs to improve the system security are listed as follows. Cainelli et al. [29] propose a DT to create resilient 5G networks in industrial systems to avoid production downtime by reducing potential disturbances and supporting changes in the production process through modifications in the use of the communication system. The DT allows for collaboration between automation and communication systems, adapting the behavior of the process in case of unforeseen events. Schellenberger et al. [33] extend the original plant with an auxiliary system that does not add additional delay into the system. The auxiliary system is designed as a linear discrete-time system with similar dynamics of the original system and that is capable of attack detection. For this detection strategy, a model of the overall system dynamics and the switching signal of the auxiliary system are needed. The residuals of the Luenberger observer are then monitored for deviations from zero, which indicates an attack. Salvi et al. [32] created a DT to improve the resilience of critical infrastructures. It enhanced the response capacity to improve the response time and reduce the impacts of attacks on power systems. The authors focused on incident prevention and response management. Similarly, Saad et al. [31] address security in microgrids. Their proposal builds the DT mathematically to protect individual and coordinated attacks. The approach detects and mitigates false data injection and Denial of Service (DoS) attacks. Huang et al. [30] propose an anomaly detection framework with real-time monitoring for industrial systems. They use distributing supervised machine learning for data processing and for making decisions between the physical layer, edge layer and cloud layer. Additionally, Sousa et al. [34] present an approach to design DTs to secure critical infrastructures. They use high-fidelity replicas of Programmable Logic Controllers (PLC) to mitigate DoS attacks that use flooding and amplification. Xu et al. [35] present a DT using a chi-square detector that prevents adversaries that learn the system dynamics to avoid detection. The approach also proposes secure estimation against stealthy estimation attacks and control for CPS using a game theory mechanism. Xu et al. [36] also present aDT for anomaly detection, but they proposal continuously and automatically building theDT with live data from a CPS. This creates a Generative Adversarial Network (GAN) to capture the temporal and spatial characteristics of the input data and recognize adversarial samples.

*Monitoring and Prediction*—The PO is monitored by sensors that collect and store data in real time. The goal is to process and use such sensed data to anticipate events. This is beneficial to control the PO and also to organize the working teams, create better synergies and use their time efficiently, which leads to a greater productivity. Moreover, the industrial sector normally requires evaluations of multiple scenarios to make decisions at the business model level. This evaluation is useful for managing risks and costs and for forecasting demand.

In [38], Barbie et al. design a DT approach to develop Ocean Observation Systems as autonomous robotic networks. It uses the Reference Architectural Model Industry 4.0. The DT provides a visual representation of synchronized commands and allows evaluating different scenarios in the virtual environment. It allows detecting errors and reduces the impact of mistakes made by an operator of that ocean observation system. Booyse et al. [40] propose a DT for system health monitoring. The approach aims at detecting and diagnosing system problems and predicts maintenance based on unsupervised deep learning. The DT is constructed using deep generative models which learn the distribution of healthy data directly from operational data at the beginning of an asset’s life cycle to produce an estimation of asset health. It learns a probabilistic representation of the real-world asset, from which it is possible to sample data from the current operational conditions and determine healthy data. Bhatti et al. [41] propose a real-time fault detection and diagnosis mechanism for industrial robot actuators. The DT monitors the system, creates an alarm and makes diagnoses as soon as electrical faults occur in the machine. Bartos et al. [39] present a DT for water management to prevent flooding and improvewater quality. It combines sensor data with online models to understand and control the system dynamics. It simulates and estimates the state of drainage networks in real time. Another example of using DTs to monitor a system is presented by Modoni et al. [42], who built a DT to ensure the quality and metrology of a micro manufacturing system, i.e., the manufacturing of compact and tiny devices that are wearable or that can be inserted into the body. The DT continuously monitors the in-line parameters of the micro production process by mirroring the physical process to compare it with analytic models and supply specific variations of parameters, so as to keep them in optimal conditions always. Moreover, predicting systems’ failures helps to better schedule the maintenance. For example, Angeliu et al. [37] built a DT to analyze building structures and optimize restoration works on buildings. Moghadam et al. [43] monitor wind turbines using a DT. For that, the DT is built with a torsional dynamic model that uses online measurements and fatigue damage estimation to calculate the remaining useful life of the system. The monitoring allows estimating the system load and stress in the different components to feed the degradation and fatigue models of each component. Errandonea et al. [52] review how DTs can be used to improve different types of maintenance, such as reactive, preventive, predictive, prescriptive and condition-based maintenance.

*Development Improvement*—Another application of DTs is to improve the design of a product and test their functionalities. Traditional product design requires a functional analyst to identify product design problems and improvement. Using a DT, it is possible to analyze large quantities of information and make more precise design decisions, which creates better product innovations. For example, Dong et al. [44] propose a DT with this objective. It allows for analyzing and improving the product design process. The DT creates a product redesign method using the functional backtrack obtained from models. Fedorko et al. [45] propose a DT for testing the properties and characteristics of a physical object, i.e., for physical experimentation. The objective is to overcome the limited possibilities to physically experiment with convoy belts and reduce the time required to do that.

In this line, DTs present a solution to create an environment to test objects using models, without carrying it out physically. Li et al. [46] propose a DT to evolve traditional manufacturing processes to more sustainable methods that control the generated environmental and social impacts. For that, it analyzes the dynamic evolution of the whole life cycle based on a DT mapping system. The objective is to create a manufacturing process capable of reducing, reusing, recycling, recovering, redesigning and remanufacturing the product level. At the production level, the objective is to reduce energy, resource consumption, toxic waste and occupational hazards [53]. Liu et al. [47] address the problem of the traceability and unpredictability of quality in manufacturing processes using a DT. The system allows detecting the fault’s source, predicting the processing and dynamically controlling the processing quality. Sun et al. [48] work on a similar problem. They propose a DT to improve the quality control and assembly efficiency in high-precision products for aerospace, marine and chemical industries. Similarly, [54] surveys the main challenges and potential applications of DTs in product design and development areas.

*User Training*—DTs provide a platform to train operators in a low-cost and low-risk environment. Using a dynamic environment helps broaden their experiences in controlling the real system operation, especially when under maintenance, adverse or emergency operating conditions [55]. Moreover, it enhances the operators’ decision-making abilities and reduces the effects of wrong or inaccurate operations. DTs help to evolve to an Education 4.0, to create a more qualified workforce and reduce the decision-making time in the industrial sector. For this purpose, it is required to add a human–machine interface (HMI) to interact with the DT. The incorporation of this functionality with Virtual Reality (VR) and Augmented Reality (AR) is attracting attention. Some research works that propose DTs for training purposes are presented as follows. Cortes et al. [49] present a DT to teach industrial concepts such as the automation and programming of programmable logic controllers (PLC), industrial network traffic and modeling using system dynamics. Waat et al. [50] propose a DT with AR capabilities using physical geometrical models. The goal is to train operators in factory assembly tasks. Junior operators are trained at the assembly line with much less supervision from senior operators than in traditional scenarios.

#### 3.1.2. Process Planning

The design of a DT is complex and includes several parts, such as models, internal divisions, interfaces, material properties, spacial geometry and how the whole system should be assembled, among others [37]. The process planning provides a description of the process activities and the relationships among the components that implemented them. It also determines which functionalities and system properties will be modeled in the DT. The main objectives of the process planning is to clarify the process requirements, the model selection and how data will be exchanged.

In most of the cases, the DT will address a phenomenon of interest, i.e., only a part of the process will be included in the DT. For this reason, it is important to determine the features or functions that will be included in the DT. In other cases, DTs may pursue an identical copy of the PO, but this situation should be analyzed due to the fact that it may bring extra complexity and redundant information. For that, Zhang et al. [56] define a set of metrics to analyze how to define the right DT for a system. Similarly, Kutzke et al. [57] propose an approach to evaluate which subsystems and functionalities will be implemented in the DT. It chooses the DT subsystems based on a set of priority metrics for each component. The priority highlights the components that should be included because they lead to the greatest increase in the total system reliability and simultaneously represent the minimal cost set of components for implementing a DT. For that they use a model-based systems engineering (MBSE) approach and present an example for unmanned underwater vehicles.

The process planning also determines which equipment will be modeled in each subsystem. This information helps to determine the DT’s development requirements and which data should be collected from the physical process. The objective is to create a comprehensive set of data describing the physical system. For example, a system dataset may include the geometry, internal partitions, material properties, past technical interventions, environmental data, deformation measurements, etc. This data will be used to build, validate, calibrate and maintain the DT.

As a result, we obtain a detailed view of the system functions, activities, characteristics and states that will be considered. We also identify and prioritize use cases for the system, metrics and requirements, such as software and hardware constraints. For the selected functionalities, data will be collected through sensors placed on the PO and sent to the VO, where it should be structured. Hence, it is necessary to define which data will be considered, for example, rotation, geometry measurements, material characterization, dynamic properties of the system, etc. It determines also the model inputs and outputs. Then, this data is classified to build a model of the PO. Usually, a combination of behavior, structural and communication patterns are necessary to model the PO.

Data is essential for the DT. Hence, the data flows should be carefully designed. The DT should reproduce how the components of the real system interact with each other and how they exchange information. The communication patterns need to be analyzed and modeled. Another point to analyze is how the DT exchanges data with humans, i.e., how the different process stakeholders interact and use the system.

For that, Rasor et al. [58] present a technique to specify the product life cycle and support collaborative planning and specifications for DTs, considering the vision and collaboration of different stakeholders across the value chain. They also use a MBSE methodology to implement their approach. Firstly, they define the macro specifications and identify the use cases of the DT in a value chain network and outline the related life cycle phases. For each use case, they assign the involved partners and their exchange with the system’s components. As a result, they obtain abstract use cases relating to the value chain roles (managers, engineering, operators, developers, etc) and the life cycle of the overall system. Secondly, they define the micro-level specifications that formalize and detail the identified use cases from abstract to concrete. Finally, they consolidate the uses cases and define an architecture and concrete implementation requirements; they also determine data sources and the underlying IT infrastructure (IT systems, components and programming interfaces) to provide and process the data and models.

Liu et al. [59] propose a method to identify the machining processes, hierarchical data structures and evaluation methods of the process. The approach uses three layers. Firstly, a data mapping layer is used to acquire, organize, manage and map the collected data. The collected data is acquired by a sensor and organized using an object-oriented method. Secondly, the approach uses a data analysis layer in charge of the dynamic model creation, and finally, a data decision layer to evaluate the process planning based on evaluation rules. Sierla et al. [60] propose a method for semi-automatic DT specification for process plants. The approach takes the available documentation, such as PDFs and other human-readable formats, and analyzes the information to propose a DT.

The output the system specification includes the objectives, uses cases, the detail of the process life cycle, the states and metrics, the roles, the hardware and the system’s software requirements. This information is useful to plan the system architecture (cf., Section 3.2) and to build the system model (cf., Section 4).

### 3.2. Architectural Design

There is no consensus about the properties of a DT and its corresponding component’s architecture. However, a DT has at least three minimal parts [8]: a physical component (PO), a virtual copy of this component (VO) and the connection to exchange bidirectional data between them. Other authors propose extending the minimal architecture to consider more components. For example, Tao et al. [61] propose that a DT has five parts: a PO, its VO and their connections, data and services. Singh et al. [62] propose an architecture with seven layers, including: the PO composed of a control unit, sensors and actuators; a communication layer in charge of data acquisition and edge processing of data; and a security layer responsible for the secure handling of data flow. The fourth and fifth layers are data storage and modeling and optimization. The sixth layer is the service layer responsible for the development of advanced data-driven applications or standard data analytics functions. The seventh layer is responsible for providing the value-adding information to the appropriate stakeholder using data visualization. It also includes devices that enable decision making for the user and the feedback of information to the physical device, e.g., via a human–machine interface (HMI) or direct feedback to the control unit of the physical twin. This architecture puts the physical process at the center and builds the DT around the real-world functionalities. It can also be created incrementally to add new components in different implementation cycles.

In addition, the DT architecture should evolve over time to incorporate system changes, such as new components, new interfaces or new connections to other components, or adapt the system behavior. The architecture should also support multiple models (cf., Section 4) and interconnection with other DTs. For this reason, Minerva et al. [16] indicate that the architecture for industrial DTs should contain the following four layers: business, service, integration and data. The business layer deals with the processes and logic related to the production of goods or services. The service layer controls how components and services can be created, controlled and managed; it also provides DTs management and simulation. The integration layer supports the dispatching of information to all the system’s components. The data layer represents the different sources and the related enterprise systems that are integrated. As a result, this architecture is more adaptable to add changes once the system is operating.

A combination of the two previous approaches is proposed by Bevilacqua et al. [63], who build an architecture to implement DTs dedicated to managing operators’ risk in process plants. It considers the three minimal parts (PO, VO and connection), and the VO is composed of control tools, simulation tools, anomaly detection and prevention tools and a cloud platform to obtain real-time data. One interesting point is that the simulation tools can be used for core real-plant functions, but they can also include additional behavior and functional models by the creation of Mock Units. Leng et al. [64] address the problem of how to create a DT architecture capable of system reconfigurations. They propose using an Open Architecture Standard Platform to change the system at the control level (for example, to integrate a new software module), at the machine level (to add new fixtures) or at the system level (for example, to change the layout configuration). Fan et al. [65] investigate how to create an architecture to model multi-source heterogeneous information and how to create 3D visualized human–machine interactions with the DT.

As shown previously, most of the proposals use their own DT architecture, which makes it harder to integrate them with a system at different levels. For this reason, Aheleroff et al. [66] propose a reference architecture for DT as a Service in Industry 4.0. It is based on the Reference Architectural Model for Industry 4.0, also known as the RAMI 4.0 architecture. The RAMI 4.0 architecture provides a common understanding of industrial use cases, which can be adopted as a model for almost all Industry 4.0 applications. The architecture is mostly defined as a system and relationships to address the fundamental structure of elements, relationships, restrictions, and logical properties. It uses a service-oriented architecture (SOA) that provides different services and breaks down complex works into simple structured packages. As a result, the DT reference model consists of four parts: a physical layer, a digital layer, a cyber layer, and communication for data exchange among the three layers. The physical layer defines the real attributes, such as objects, assets, products, personnel, equipment, facilities, systems, processes and environments. Sensors and actuators are the two main connected components in the physical layer. The digital layer is the recording of data in raw or different file formats, such as Computer-Aided Design (CAD) or Computer-Aided Manufacturing (CAM). The digital layer is dedicated to the creation of digital copies of physical things. The cyber layer includes the cloud processing and storage for building a dynamic data model. It offers scalable and distributed computing technologies such as AWS, Azure Databricks, Hadoop and Google Analytics. The cyber layer also adds several competitive advantages, including data privacy, transparency, scalability and individualization.

In line with this clould cyber layer, Alam et al. [67] analyze how to integrate clould services into a DT architecture for CPS. They argue that integrating cloud technologies in the CPS cyber layer ensures the scalability of storage, computation and cross-domain communication capabilities. For that, they identify different degrees of hybrid computation–interaction modes and designed the interaction of the controller using a Bayesian belief network. They also include fuzzy rules to support reconfiguration capabilities.

## 4. Digital Twin Modeling

**Figure 3:**
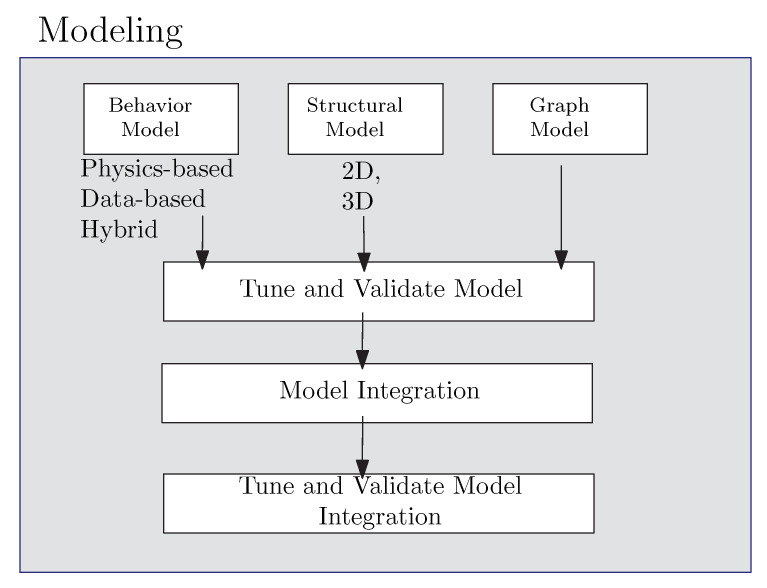
DT modeling process.

The core of a DT is the virtual models. For this reason, the most important step to build a DT is to create high-fidelity virtual models to reproduce the geometry, physical properties, behaviors and rules of the PO. In a DT, the physical real-time data may also be required to update the virtual models and simulate the physical process and its evolution. In this section, we present how to build a model for a DT. Figure 3 depicts the process.

### 4.1. How to Model a Component

To create a DT, we need to model the physical reality using abstraction. In this section, we analyze how POs are modeled to obtain their VO. The main approaches that can be used for that are classified and summarized in Table 2.

#### 4.1.1. Behavioral Models

These kind of models are a specification of the system behavior based on the physical process that the PO controls. As a result, these models refer to a mathematical or computational description of how the variables of interest relate to each other, for example, to understand how forces, acceleration, jerk, angular displacement, angular acceleration and other phenomena interact in the physical process.

The main approaches to building behavioral models include using control or data-based techniques. The control techniques consist of observing a physical phenomenon, developing an understanding of it and expressing it in the form of mathematical equations. Understanding physical process is hard and may not be possible in complex systems. However, it provides tools to reason about the system behavior, which is useful to generalize the models to similar problems and also to bound errors. On the contrary, the data-based models work as a black box and do not provide these advantages. The positive points of these approaches is that they are more flexible and can take into account new experimental data. They may also be more suitable for complex systems where understanding the physics is not possible, or even in systems where physics models cannot be applied, such as 5G networks. The models obtained by data may be biased by the dataset and the errors cannot be estimated [71].

*Control Models*—These are physics-based models, i.e., they use the laws of physics and compare simulated results with known information, represented by mathematical models [20,72]. Control is the core function of DTs built for CPS. It aims at maintaining the system at an acceptable level of operation in response to disturbances. In CPS, the physical processes provide information to the cyber components, and the cyber components control the physical processes. In order to remain consistent, real-time data from the physical process is collected using sensors. The data is communicated to the cyber components and it is used to compute the control output to send it back to the actuators to correct the physical process.

A behavior model based on control theory can be obtained using transfer functions or state-space modeling. This way, the reality is theoretically modeled using representations that relate to each possible input signal and the corresponding output signal. Using this technique, the design process starts with the differential equations that model the behavior of the physical process being controlled. Then, the transfer function can be derived from the differential equations of the process. The transfer functions and the state-space modeling are equivalent representations, i.e., one can be derived from the other and vice versa [73].

The transfer function G(s) is the ratio of the Laplace transformation using the complex variable *s* of the output Y(s) to that of the input U(s). It is represented, as shown in Equation (1), by the division of two polynomials; the numerator is created by taking the coefficients $b_{i}$ of the output differential equation and the denominator using the coefficients $a_{i}$ of the input differential equation.

(1) $$G\left(s\right)=\frac{Y(s)}{U(s)}=\frac{\sum_{i=0}^{m}b_{i}s^{m-i}}{\sum_{i=0}^{n}a_{i}s^{n-i}}$$

A transfer function with multiple inputs and multiple outputs is usually represented in matrix form, which indicates the relationship of each input and each output of the system. The state-space model expresses the differential equations in matrix form, as shown in Equation (2):

(2) $$\left.\begin{array}{c} x_{k+1}=Ax_{k}+Bu_{k}+w_{k}\\ y_{k}=Cx_{k}+v_{k} \end{array}\right.$$

where $x_{k}\in R^{n}$ is the vector of the state variables at the *k*-th time step, $u_{k}\in R^{p}$ is the control signal and $w_{k}\in R^{n}$ is the process noise, which is assumed to be a zero-mean Gaussian white noise with covariance *Q*, i.e., $w_{k}∼N\left(0,Q\right)$. Moreover, $A\in R^{n\times n}$ and $B\in R^{n\times p}$ are, respectively, the *state* matrix and the *input* matrix. The value of the output vector $y_{k}\in R^{m}$ represents the measurements produced by the sensors that are affected by a noise $v_{k}$, assumed as a zero-mean Gaussian white noise with covariance *R*, i.e., $v_{k}∼N\left(0,R\right)$, and $C\in R^{m\times n}$ is the output matrix that maps the state $x_{k}$ to the system output.

DTs require replicating the states of the physical process within a CPS in functionally equivalent virtual replicas to mirror the internal behavior of the system. To solve this issue, Eckhart et al. [74] analyze how to passively replicate the program states of devices to obtain a virtual representation of the CPS during its operation. They propose an approach that identifies stimuli on the system’s specification and then replicates them in a virtual environment. This way, the stimuli triggers state transitions and different data sources, such as network traffic or system logs, can be used to identify the stimuli, replay it and synchronize it between the DT and the PO. Similarly, Schellenberger et al. [33] propose a DT to detect attacks in CPSs. In this case, the approach is based on Luenberger state observers [75] to estimate the state of the system based on observations of its inputs, outputs and a mathematical model that describes its dynamics, i.e., an observer is a continuous-time dynamical system that takes as input the measured input and measured output of the plant and produces an estimate of the state of the plant as output.

*Data-Dependent Models*—These are based on data structures that retain all the variables describing the reality at the level of abstraction chosen. With the data supplied by the PO, it is possible to build the VO with the help of Artificial Intelligence (AI) open-source libraries such as TensorFlow [76], PyTorch [77] or OpenCV [78]. This approach is based on the assumption that since data is a manifestation of both known and unknown physics; by developing a data-driven model, one can account for the full physics [71].

To build this type of model, it is required to develop a four-stage process which involves data generation, data collection, data pre-processing and data analysis through AI algorithms. The data generation is strongly based on sensors that collect information from the PO. Multiple tools for data collection also exist that can support real-time data collection, such as Apache Kafka [79]. After that, the system will have a huge amount of data. Hence, it is required to pre-process it to ensure the quality and completeness. It is also necessary to compress it and summarize it. The process requires evaluating the relations between variables and detecting noise. This process of data engineering also includes cleaning the data to correct or remove corrupt and inaccurate data. For that, operations such as filtering, handling missing or erroneous values and removing redundant and duplicate information are used. Furthermore, data integration, data transformation and data enrichment are also parts of the data engineering process. Apache Spark [80] is one useful framework for memory-based data processing. As a result of the operations, the pre-processing increases data accuracy and saves computational cost.

*Hybrid Control–Data Models*—As explained previously, both control and data models have advantages and disadvantages. The hybrid models try to obtain the strong points from both design techniques [81]. The use of a control model ensures physical interpretability, which is very useful, for example, in engineering systems. Machine learning models are very well adapted to data and are suited to real-time applications [82].

For example, ref [83] proposes the integration of physics-based models with machine learning to design a DT to predict structure damage. This strategy allows the use of an interpretable model (physics-based) to build a fast DT (machine learning) that will be connected to the PO to support real-time engineering decisions. In addition, ref [84] shows how to build a hybrid DT model of a heater in a water process system. The work details the steps for updating the physical model and process system using data-driven models of the process equipment. This way, with the help of history data to teach ML models, the DT can be continually improved over time. Chakraborty et al. [85,86] also propose a hybrid control model for linear single-degree-of-freedom structural dynamic systems evolving in two different operational time scales. The approach uses a physics-based model for data processing and response predictions, and a data-driven machine learning model for the time-evolution of the system parameters.

*Other Modeling Techniques*—Some modeling techniques do not use the physics of the system but the relation of the components. For example, it is possible to use graph models to represent communication models or knowledge-based models that require having an expert to analyze the system and manually design a modelization of the characteristics and behavior. Other methods include the one proposed by Dai et al. [68], who proposed an ontology-based method to model as-fabricated parts. They argue that this methodology provides a standardized process to create DTs. Through this modeling technique, engineers may perform evaluation and optimization of machining processes. To create the DT, the model encapsulates the physical data and information relationship with its external environment. They use a model dependent on realism and it is based on the belief that all we can know about reality consists of networks of concepts that explain observations by connecting the concepts with rules to define models. The realism also suggests that we cannot know the reality as it is, but only approximations of it represented by models. This way, a rational information model can represent critical concepts and their relationships. Additionally, Pylianidis et al. [69] propose creating DT models using simulation-assisted ML algorithms. They use process-based models integrated with ML to adapt the resulting model to the input data. The process-based model aggregates data to a lower resolution to mimic real situations and develop the ML models using a fraction of the process-based model inputs.

#### 4.1.2. Structural Model

This model defines a structured description of the connection and assembly relations among the structures that perform the functions and behaviors. The interrelation of the structure is the foundation for the transferring and transformation of the material, energy, information and motion behavior of the system. The structural model usually includes topology definition, layout planning and buffer designing [13].

A Physical Model enables simulating the physical properties and loads, analyzing phenomena such as deformation, cracking and corrosion [10,70].

A Geometric Model reflects the geometry, shapes, sizes, positions and assembly of machine components, the kinematics, the logic and the interfaces of the real system [87,88]. For instance, 3D modeling is one of the techniques used to represent system geometry. It is the process of developing a mathematical representation of the surface of an object. The 3D models can be constructed by a 3D scan of the object, or through specialized software using equations, and are finally represented in terms of curves and surfaces [71]. Image-based methods also offer a good alternative to geometry measurements, compared to scanning techniques. The image-based approaches permit reconstructing the geometry using image processing algorithms based on digital photogrammetry. In addition, they can be complemented with data that describe the internal structure of the object that can be obtained by classical methods of inspection, thermal imaging or radar techniques, which allow for investigating a physical structure in more depth [37]. Anbalagan et al. [89] explain how to create Digital Geometry models using Computer-Aided Design (CAD). They discuss CAD modeling and manufacturing simulation methodologies in a virtual environment. The objective is to create geometric models useful for DT design.

### 4.2. How to Integrate the Component Models

DTs are the integration of complex interconnected models, i.e., different models are integrated to create a more complete view of the system. The integration has to consider different aspects, such as:

1. How different components interact with each other to create more complex systems. This means that models interact with each other to represent the PO behavior. It should be considered that decisions made by some models can modify or invalidate the conclusions of other independent models. As a result, wrong or conflicting results may exist if models do not share information and make coordinated decisions;
2. How the DT interacts with the physical world, i.e., a DT makes decisions that directly or indirectly impact the physical process. It may be difficult to delimit the physical impact a priori. For that, the digital components should propagate the decisions using the physics laws of the PO to evaluate the effects of these decisions and the inconsistencies that may arise.

In large and heterogeneous projects, the interactions and dependencies can produce errors if they are not correctly modeled. This means that even when each model is independently correct, their correctness within the whole system may depend on its relation with other models. For this reasons, model integration is a complex and important part of the DT building process. Table 3 summarizes model integration techniques.

*Hierarchical Integration*—This approach builds complex systems by integrating smaller and simpler components. This way, unit models can be organized hierarchically to obtain a model of the whole process and understand how the different pieces work together. Complementary models can also be integrated horizontally to obtain a wider view of one component, for example, a control-theoretic model of the physical behavior and a geometrical 3D model.

In this line, Tao et al. [90] propose a hierarchical integration where the unit level is the smallest unit of the process, for example, a single piece of equipment (such as a machine tool or robot arm), raw material, components with sensors or even environmental factors.

At the system level, multiple unit levels are interconnected and inter-operate to enable a wider range of data flow and resource coordination. For example, a production line, a shop floor, a factory or even a complex product (such as an aircraft) are examples of system-level DTs. The virtual models of a system-level DT are formed through the integration and collaboration of multiple unit-level models.

In a system of system-level DTs, there are cross-system interconnections, interoperability and collaborative optimizations between the system-level DTs. For instance, a system of system-level DT may focus on enterprise or cross-enterprise integration to provide commerce, supply chain and manufacturing cooperation [96]. It can also integrate various stages of the product life cycle, such as design, production or remote maintenance. Borth et al. [91] also work on the idea of a system of systems. They discuss integration challenges as well as strategies and architectures to address the integration, for example, the use of modular approaches based on causal thinking to structure the inner DT models or the integration of reflection to integrate self-awareness in the DT regarding its performance. They analyze the system-of-a-system-of-DTs perspective using, as an example, smart grids and smart building applications.

*Collaborative Integration*—A collaborative information model defines how different components interact and simulates the collaborative behavior among several assets [11]. In addition, decision-making models make the model capable of evaluating, reasoning and validating. They consist of variable inputs, algorithms and a collection of constraints and rules. They also include rules of constraints, associations and deductions. They store and analyze the running status data and then make decisions using machine learning algorithms.

For example, Autiosalo et al. [92] propose an open-source git-based server software to build DT webs, i.e., to build networks of DTs with a similar structures to that of the World Wide Web. The DT Web is created with a combination of standards and technologies. The objective is to allow DTs to be interlinked and exchange information in the same way as their real-world POs. Cimino et al. [93] propose a paradigm called the Digital Multiverse to comprehend DT interpretations at the data integration level and also by establishing and enforcing consistency rules. For that, they create relations that involve both data, models and the different system life-cycle management methods. For example, they define viewpoints based on roles such as plant designer, control engineer, data scientist, maintenance manager and so forth. The models and data are bound together by relations, so that no operation on any view on the different DTs’ perspectives can lead to inconsistencies. They formally relate not only data to data, but also data to models and models to models perspectives. Eramo et al. [94] propose a conceptual modeling framework to integrate, synchronize and manage different models, data sources and their relations in DT. The framework details the core parts of DTs and describes which parts need to be provided, interconnected and integrated to achieve different DT utilizations. Sahlab et al. [97] propose using knowledge graphs to relate complex and dynamic digital models. They argue that the inherent extensibility, adaptability and semantic expressiveness make knowledge graphs suitable to manage models and enable reasoning to gain new insights. Zheng et al. [95] propose a DT for smart manufacturing using three models, a digital model, a computational model and a graph-based model. The tri-model works concurrently to simulate real-world physical and digital behaviors and characteristics. The digital model is a structural representation of the system, for example, using numeric values such as dimensions, equations and their relations to control the geometry. Their computation model is a behavior model that includes the laws of physics that govern the PO. Finally, the graph-based model establishes the relationships among physical systems and within them, considering both the structured information of the objects and the unstructured data from the context. This way, they create a multi-physics model that captures the system behavior, the effect on the structure and geometry and its interaction with the environment.

## 5. Digital Twin Development

The next step in the DT building process is to implement it. In this section, we review protocols, tools and standards to develop and synchronize the PO and its VO.

### 5.1. Communication Protocols and Data Synchronization

The network is vital for communicating the PO and its VO. The connection allows for collecting real-time data and also exchanging control commands. The PO and its VO exchange data bidirectionally, i.e., data collected from the PO is used to update the states maintained in the virtual representation. Moreover, data from the virtual representation goes back to the physical system. As a result, the insights and decisions generated in the virtual representation provide feedback to the physical system. In general, the PO-to-VO communication requires three steps [20]: (1) collect the relevant information, including the direct measuring of the physical reality; (2) interpret the data to a form that is consistent with the level of abstraction—this may include several steps, such as data processing, data curation and data conversion; and (3) use the data to update the states of the system. This may require information fusion, i.e., the use of data from multiple sources.

For this reason, we analyze how different research proposals connect both components and manage the data exchange.

Each component of the system requires a special type of connection. For example, the sensor and actuators are limited-resource devices that send data at a specific frequency or when new events happen. Hence, low-power consumption protocols, such as 6LoWPAN, Bluetooth, ZigBee or LoraWAN, are more adapted to them. However, for data exchange, it may be necessary to use protocols such as MQTT, CoAP or AMQP. The data from numerous sensors is collected by a gateway that aggregates the information, which is sent to the DT. Connections between sensors and gateways can take place using multiple technologies depending on the type of physical system. Many commercial solutions have focused on providing solutions to communicate IoT devices and enable data exchange and processing—for example, Amazon Web Services (AWS), Microsoft Azure IoT Suite, SAP cloud platform, Salesforce IoT, Oracle IoT, Cisco IoT Solutions, IBM BlueMix cloud and Bosch IoT [71].

Synchronization and consistency issues limit the number of possible VOs connected to a PO. However, in case of multiple VOs, the communication between both components can be implemented using a Publish–Subscribe technique, where the PO is considered the information publisher and the VOs subscribe to receive the information. This communication pattern is also useful to communicate only interesting events, instead of periodic updates. Protocols such as MQTT and platforms such as Apache Kafka support this kind of communication.

The data connection in critical DT applications is required to be in real time. As a result, latency is a critical factor for the user experience and the system functions. For example, in robotic surgery, the action of a surgeon should be performed in real-time with no latency. Otherwise, the result will be inaccurate. To reduce the communication latency, there are three main approaches [98]. The first one is to deploy high-speed network links using, for example, optical fiber cabling for wired connections or 5G technology for wireless connections. The 4G networks decrease their performance as more devices share the limited radio-frequency spectrum. However, 5G technology has a wider frequency range to accommodate more devices that require communication at higher frequencies [71]. The second option is to use data compression to reduce the amount of data to be transmitted. This can be implemented with compression techniques, data filtering and also AI algorithms that process, clean and aggregate the data before sending it. The last option is to move the calculation and data processing near the physical process, for example, using edge or fog computing capabilities to support the processing requirements of the DT. This way, it is possible to process the information faster than in a full cloud-based analysis.

Other aspects to consider is that the connection should be bidirectional and both components must keep consistent data. Hence, a means for keeping both components updated and the interfaces to exchange this type of connection should be designed. For that, Platenius-Mohr et al. [99] analyze the communication requirements that a DT should meet, such as supporting different formats, including a variety of source and information models and the semi-automatic definition of syntax and semantics, among others. They also present an approach to improve the current lack of interoperability between DTs of different companies that require information exchange. To do so, they transform the information to other formats using a customizable mapping model. The mapping model is customized and interpreted on demand. The solution enables file- and API-based information exchange in a bidirectional way. Jiang et al. [100] propose a framework to integrate and improve the data exchange between the PO and the VO. It focuses on the device acquisition layer and the remote system, which can be composed of multiple partial DTs logically built with different functions. They also create a collaboration between edge and cloud resources to improve the cooperation of different components and data fusion. Barbieri et al. [101] propose a methodology to enable real-time coupling between the DT and the controllers in a physical plant. They propose a formal modeling language and a design pattern to generate a standard interface for the communication between PLCs and digital models within a DT architecture to facilitate the exchange of information. Li et al. [102] include QoS consideration in the communication and system modeling. They propose a framework combining semantic resource modeling with real-time industrial object transmission to improve the performance of DT. They consider the interoperability, scalability and time delay as QoS factors.

Finally, we must consider the security of the DT interconnection. In this direction, Feng et al. [103] discuss the security problems of wireless communication in a DT and analyze how to secure the communication channel between the PO and VO. They propose an approach to reduce interference attacks in the communication network. They use Attribute-Based Encryption to meet the security and overhead requirements and they use an access control policy to encrypt a random key, as well as a symmetric encryption scheme to hide it.

### 5.2. Experimental Platforms and Tools

Existing frameworks can accelerate the DT development process. For this reason, in this Section, we review the existing efforts to provide tools to implement DTs as well as the proposed experimental testbed to test the DT approaches.

*Development Platforms*—Some initiatives have developed platforms to build, test and benchmark DTs. For example, Bonney et al. [104] present an open-source, modular, and system-independent framework to build DTs using Flask Python. The platform uses HTML web pages as the interface between the DT simulations and the user. The tool can deploy the DT with multiple accessibility options to accommodate a wide variety of intended uses. It can also be itegrated with third-party simulation tools and can be configured with model data, such as geometry and material assignments, among others. Borghesi et al. [105] propose a reference architecture for DTs in IoT platforms. They analyze the requirements for the management of industrial DT scenarios and different use cases that are implemented in the DT platform. The architecture allows an implementation using diverse technologies to fit the application use cases, technical requirements and be integrated with existing systems. In [106], Bolender et al. present a modeling framework for self-adaptive manufacturing. It supports modeling domain-specific cases, describing rules for case similarity and case-based reasoning within a modular DT. They propose automatically configuring DTs based on modeling tools to improve the manufacturing times, reduce wastage and contribute to better sustainable manufacturing. For that, the modeling framework provides multiple interrelated modeling languages and integrates them into our model-driven architecture for DTs. Eclipse Ditto [107] is an open-source framework for IoT DT software implementation. It mirrors physical devices, provides services around the devices and keeps the PO and VO components in synchronization. FIWARE [108] is a middleware for managing data in a DT. It is a framework of open-source components, known as Generic Enablers (GEs), for facilitating the development and implementation of smart solutions. Commercial solutions to develop DTs also exist, such as the ANSYS Twin Builder, iTwin Bentley, Siemens, MapleSim, General Electric and Oracle Digital Twin platforms.

*Experimental Testbeds*—Previous research has proposed testbeds to test new approaches. For example, Kamath et al. [109] propose an open-source testbed for building Industrial System DTs using Eclipse Ditto. It includes real-time data acquisition, virtual representation, analytics and visualization.

Albo et al. [110] present a framework with modular software to build a 3-DOF robot DT. It uses CoDeSys [111] to implement Programmable Logic Controller (PLC) logic and a Human–Machine Interface (HMI) for controlling the system. The robot node is controlled using a programmable script compatible with several programming languages such as Java, C, Python and MATLAB. MATLAB and Simulink [112] are used to model the multidomain physical behavior. They verify signal properties and simulate device logic. They also send data to the PLC to control the motion of the robot kinematic model. Webots [113] enables importing CAD models, either as pure geometrical shapes or as formats containing additional information, such as the kinematic relation between solid bodies with material properties. The modular setup enables isolated development work across the different levels of control, from the PLC logic down to the kinematic properties of the digital model, focusing on a single point of change and reduced interconnected constraints. The implementation can be used in other domains since model-based libraries within Simulink can provide solutions to other type of behavior models using, for example, Simscapes Fluid, Driveline, or Multibody.

Eckhart et al. [114] proposed a framework to create and execute DTs. The approach automatically generates a virtual environment from the specification and matches their POs. They include two main modes of operation. First, a simulation mode that operates independently of the physical environment to monitor and explore a virtual clone without risk, and second, a replication mode, replaying events from the physical environment for visualization and analysis. The framework also has multiple modules that can be activated on demand, such as monitoring, security analysis and intrusion detection. This framework was proposed with the objective to improve CPS security properties. For this reason, it is possible to activate diverse security and safety rules to be automatically monitored by the DT. In addition, security testers can attack the system security using the virtual replica of the production setup.

## 6. Discussion

Other review surveys have been proposed in the related literature. Some of them focus on analyzing the DT definition and its uses. The term Digital Twin has been interpreted differently by different authors. Wagg et al. [6] survey the history of DTs, their definition and their objectives; the main focus of the paper is on DT applications, but they also discuss how to create a model using physics and how to verify it analyzing a case study. Barricelli et al. [7] also focus on the three following questions: what are the definitions of DTs published in the literature? What are the main characteristics that a DT should present? What are the DT domains of application? Lu et al. [115] also review the connotations of DTs, application scenarios and applicable standards. Hribernik et al. [116] survey the required properties and requirements of a DT. Then, they propose a roadmap towards the creation of autonomous, context-aware and adaptive DTs.

With a focus on manufacturing industry, Cimino et al. [117] review which are the applications of DT. They present the automation pyramid in CPS systems and the existing application for DT. Finally, they explain how they build a DT for energy consumption monitoring using Matlab and Simulink. Davila Delgado et al. [118] survey how DTs are structured in manufacturing and how they function. They analyze the difference between DTs, cyber–physical systems (CPS), and building information modeling (BIM). They review how conceptual models, system architectures, and data models work. Their main contribution is the structural and functional descriptions of DTs in manufacturing. These two last points are analyzed in our survey and they are included as part of our methodology for designing and building DT systems. Regarding the DT definition, properties and uses, we present a short introductory context, but this is not the main focus of our research due to other works in the literature having already analyzed it.

Other studies also how DTs may be applied for specific uses, for example, Eckhart et al. [51] survey how DTs can be used to improve the security of CPS. Malik et al. [119] explore the opportunities of using a DT to address the complexity of collaborative robots and human–robot interactions. Leng et al. [13] analyze DTs for manufacturing systems and how industrial systems have evolved from Industry 1.0 to the new Industry 4.0, which uses DTs. For that, they analyze how the design steps and supporting technologies have evolved over time. Lo et al. [54] review DTs created to improve the product design and development process. Henrichs et al. [120] review DT applications in the food industry. They analyze how DTs can be used to solve problems, such as how to feed a growing population, how to avoid food loss and waste and how to improve the existing inefficient production systems. Kantaros et al. [121] review current trends and limitations in 3D printing and the implementation of DTs for additive manufacturing. DTs can also help to improve the fabrication process of 3D-printed objects. As a result of their review, they point out that the progress of DTs is still limited by the lack of DT developing methods, which is the main subject of our review. Wu et al. [122] review DT applications for ultra-precision machining to improve the performance and build processes of highly accurate technology components. Semenkov et al. [123] analyze how DTs can assess Cyber–Physical Systems and provide a precise evaluation of the system design and characteristics. Steindl et al. [124] analyze architectures and frameworks to develop a technology-independent Generic DT Architecture aligned with the Reference Architecture Model Industry 4.0 (RAMI 4.0). Neethirajan et al. [125] review the application of DTs in the livestock farming sector to improve large-scale precision livestock farming practices, machinery and equipment usage and the health and well-being of a wide variety of farm animals. All these aforementioned works survey interesting DT application scenarios. In our work, we tried to provide some more focused but realistic uses of the DT approach in order to extract a concrete methodology to conduct the building process of DTs.

In a similar vein, other literature reviews analyze the interaction and integration of DTs with complementary technologies. For example, Rathore et al. [126] survey the role of new enabling technologies such as AI and Big Data in DTs. They aim at determining the relationship between AI, ML, big data, IoT and DTs, as well as the tools required for the creation of AI-enabled DT and the criteria to build successful DT-based systems. Perno et al. [127] also survey enabling technologies of DTs and the existing barriers or limitations that they create to continue developing DT. These topics are out of the scope of this paper. Finally, Schroeder et al. [128] analyze how to build a DT architecture. They also present a case study of a system with four valves that operate automatically. Similarly, Minerva et al. [16] also review DT properties, architectural models and supporting technologies. In this paper, one of the methodology steps is to build the system architecture; we analyze some of the main proposals but do not focus too deeply on this point.

In all the aforementioned literature reviews, the difference between digital models, digital shadows and DTs creates ambiguity, and they are sometimes used as synonyms (cf., Section 2 and definitions therein). The main issue is that arriving at a fully bidirectional connection between the PO and the VO is not easy. In fact, DTs require real-time interactions to achieve their best potential. How to bring all the data together, despite latency, storage limitations and data format boundaries is a challenge that needs to be evaluated during the building process.

Digital models are a key component of DTs. However, to obtain all the benefits of the DT’s feedback and PO management enhancement, it is required to have a holistic view of the system and its interconnectivity. Indeed, building DTs is currently a complicated task. It requires the integration of diverse technologies, the interaction of different systems and the development of complex models. All the technologies, the vast amount of data generated and the required activities in the design, modeling, and development phases (cf., Section 3, Section 4 and Section 5) show that DTs are difficult and expensive to create. There are many advantages and potential applications of DTs (cf., Section 3.1), and industries are considering how to change their businesses to adapt to future tendencies and how their business models are relevant for the entire life cycle of their asset. In this context, the adoption of DT approaches requires a more efficient and easier building process.

The evolution of DT-required technologies, such as AI, 3D modeling, cloud computing, IoT and big data, will help to achieve these tasks easier, for example, with techniques to capture the real properties of the physical world and translate them into the digital world, as is the case of laser scanning methods to build 3D models from real objects. Hence, defining and improving the techniques and tools to design and create a DT with less effort and in a faster manner are essential to the development of this promising technology.

In terms of perspectives for future research, as a result of the work initiated in this review, there are several directions for improvement. This creates a great opportunity for researchers to find solutions to address such limitations and reduce the number of open problems. We summarize next some of the research gaps, limitations and trends to provide further discussion and suggestions to improve the development and adoption of DTs. Sume further (but challenging) ideas are described next.

## 7. Open Challenges

In the following, we summarize some research gaps, limitations and trends to provide further discussion and suggestions to improve the development and adoption of DTs.

*Models*—A DT requires models with complex structures and behavior to reflect the real-time operations and dynamic behavior of POs. Most of the research on DTs focuses on how to model the system. However, there are still some open issues. Firstly, a DT integrates a number of different models and processes which require verification and validation individually and as a comprehensive system. How to prove that the model is accurate and correct with respect to the real copy has not been explored enough. In addition, modeling the digital copy is complex and requires a large amount of data. Hence, the existing modeling techniques may be hard to apply to old systems, such as industrial factories. The process to build a DT is easier if IoT technology and big data are already available in the system. Another issue is that complementary and context models need to be integrated in combination with other models of system behavior. This provides a more precise model of the real system and its interaction. An analysis of how to connect and create collaborations between individual models is still required.

*Integration of DT information into the system control*—The innovative aspect of DTs with respect to simulations is the bidirectional information exchange between the PO and the VO. However, in most of the proposed DT applications, the connection is unidirectional or they do not mention how they connect the DT to the control system of the PO. In a typical system, the control system makes decisions to manage the behavior of the PO and to determine how to merge the models from the control system, and the DT information may not be trivial. It is also needed to specify how the control system will evaluate and predict the new commands decided with the DT external information.

*Secure DT Design*—It is necessary to further analyze how to build a secure DT. Most of the existing modeling approaches are based on AI techniques, either to create the initial model or to make the physics-based model evolve over time. AI techniques assume benign data. As a result, they are vulnerable to malicious modifications of the the training data (i.e., poisoning attacks) or test data (i.e., evasion attacks) [129,130]. DTs require correct and reliable information. Hence, such attacks can have a great impact on the created models and, in consequence, on the decision the DT sends to the physical process. However, physics models have similar vulnerabilities [131,132]. Data injection attacks can make the controllers lose visibility of the process control or even calculate incorrect commands by intercepting the network communications and sending malicious data. Hence, even when the model is accurate and correct, how to protect the system from attacks against the modeling techniques or the data used to generate them is also unknown. Some authors [133,134] have proposed countermeasures to face AI attacks. However, if these algorithms are incorporated in critical infrastructures, further analyses should be required to evaluate if they are efficient and useful for DTs. Even when there are no attacks, the DT software may not be reliable enough to handle autonomous control systems, as showed by Lin et al. [135]. They provide a risk assessment for DTs in autonomous control systems, in particular in nuclear power plants. They argument their concerns about whether the information from a DT is credible to support decisions of high consequence and analyze uncertainties that may put the system at risk. For example, failures and un-analyzed events can have consequences on plant response.

To solve the potential cybersecurity problems, some authors propose using Distributed Ledger Technologies (DLT), such as blockchain, to prevent the mentioned attacks. For example, Lee et al. [136] propose developing an integrated DT and blockchain framework for traceable data communication. The blockchain authenticates and makes all data transactions traceable, adding confidence to the data. Mandolla et al. [137] also use blockchain to secure the data generated through an end-to-end manufacturing process in the aircraft industry. The DT represents the manufacturing suplay chain and they show the potential of DLT for addressing each of the components of the system. These solutions are promising, and a further analysis of performance and benefits will be beneficial to improve trust in DTs applied to critical systems.

*DT Data Analysis*—The PO and VO may generate a huge quantity of data. However, it is important to ensure the data are not of inferior quality. The data needs to be, firstly, sorted and cleaned to ensure this before using them in the AI algorithms. Then, the data needs also to be stored and secured. The data fusion and analysis with incomplete or inconsistent data is also needed. The system should be scalable to ensure that the necessary storage and processing capacity is available. In addition, it may be hard to visualize and interact with the DT since it may be difficult to know which data will be useful. How to efficiently process, analyze and store large volumes of data in a DT is still an open challenge to ensure the required performance and scalability. In this line, it is important to determine which data are enough and relevant to the DT’s function to reduce the generated data while preserving the important information.

*Implementation Tools*—The development of DT tools is vital to make the building process faster and to improve the re-usability of standard DT solutions. Modular approaches may help in the construction of flexible solutions by facilitating new applications. Common tools and protocols will also help to integrate the DTs of different providers.

During the analysis of the literature, we found few contributions regarding model evaluation, DT testing and the tuning and calibration of the solution. Further contributions about methodologies, approaches and also tools to verify and validate will help to improve the truthiness and reliability of the DT information and decisions.

*Standardization*—The definition of standards and communication protocols will ensure the interoperability of DTs with their physical object and also among different DTs, as mentioned in the previous point. The development of the interoperability standards is important for the evolution and adoption of the applications. Reference models, architectures and protocols are also necessary to work towards a full integration of DTs within an ecosystem of diverse systems and DTs.

*DT Connection*—A DT may require to connect a huge number of heterogeneous components using a low-latency connection. The ability to collect, aggregate and exchange data with different components and suppliers represent, today, many interoperability challenges, especially if the synchronization has real-time constraints or proprietary interfaces. It may also be hard to integrate data from legacy machines. Another factor to consider is the possibility of heterogeneous networks with diverse devices that may be hard to integrate. As a result, the interoperability to allow different assets to work together also needs to be considered as part of a large architecture. The DT environment is usually large distributed networks. How to handle these kinds of networks, model them and scale them is also an issue to be analyzed. The data synchronization between the PO and VO must be a secure connection, as discussed previously. It should ensure a highly available synchronization with integrity and confidentiality protection.

## 8. Conclusions

Digital Twins (DTs) open new possibilities to optimize, monitor, simulate, predict, diagnose and control the behavior of physical process. They provide information to create new business models and decision support systems and optimize operation. We have presented a review of the recent literature with a specific focus on DT construction. We have covered and summarized the methodological approaches to build DTs published in the recent scientific literature. Based on such results, we have analyzed the needed steps to methodologically build a DT, covering phases such as design, modeling and implementation.

We have addressed the following research questions: (1) how to model physical objects into the virtual objects underlying the DT concept; (2) how to structure the architecture of the DT; (3) how to synchronize real-time data between both physical and virtual components of the DT; and (4) what are the main challenges in developing DTs. We have also discussed the differences of our work with respect to related work, as well as open issues and future research lines to address the challenges that still exist in this research field. Thus, we have covered critical challenges and evolution trends in the field.

We have also concluded that the growth of DTs will be based on complementary technologies, such as AI, IoT and big data analysis. Network connectivity will also be of high importance, since it enables the data transfer from the physical object to be processed by the virtual counterpart. We consider that further work must target the construction of secure DTs, exploring feasible ways of hardening the protection of sensed data in order to create reliable systems that can be suited to critical processes. Works toward standardization must also cover current limits in the integration of DTs and their potential uses to create more complex simulation systems.

## Figures and Tables

**Table 1:** Sample requirements for the specification of a DT.

Optimization			
**Reference**	**Application**	**Modeling**	**Function**
An et al. [25]	Aircrafts	Control Models	Reduce methane emissions.
Bhatti et al. [24]	Electric cars	Hybrid Model	Increase energy efficiency and reduced greenhouse gas emissions.
Bottani et al. [26]	Industry	Model-based	Optimize and prevent high-risk events for a beverage pasteurization system.
Guo et al. [27]	Industry	Structural	Optimize the layout of assembly positions in the manufacturing industry.
Gonzalez et al. [28]	Industry	Control Models	Evaluate, control and correct a transportation system.
Stan et al. [22]	Industry	Data-based	Distribution planning, activity scheduling, resource allocation, resource monitoring, process control and maintenance of resources.
Wang et al. [23]	5G Networks	Data-based	Manage 5G slicing efficiently in terms of cost and performance.
**Security**			
**Reference**	**Application**	**Modeling**	**Function**
Cainelli et al. [29]	5G Networs	Communication	Design resilient 5G networks for industrial systems to adapt their behavior in case of unforeseen events.
Huang et al. [30]	Industry	Data-based	Detect anomalies with real-time monitoring.
Saad et al. [31]	Industry	Control Models	Improve resilience in microgrids against coordinated attacks.
Salvi et al. [32]	Industry	Data-based	Improve attack response and minimize the impact in power systems.
Schellenberger et al. [33]	Industry	Control Models	Detect cyber–physical attacks in CPS.
Sousa et al. [34]	Industry	Data-based	Mitigate DoS attacks on critical infrastructures.
Xu et al. [35]	Industry	Control Models	Secure estimation and control for CPS attacks.
Xu et al. [36]	Industry	Data-based	Live data analysis to detect attacks in CPS.
**Monitoring and Prediction**			
**Reference**	**Application**	**Modeling**	**Function**
Angjeliu et al. [37]	Buildings	Hybrid	Optimize restoration works.
Barbi et al. [38]	Ocean Observation	Data-based	Analyze executed actions and evaluate different scenarios in the virtual environment.
Bartos et al. [39]	Drainage networks	Control Models	Water management system to prevent flooding and improve the water quality in real time.
Booyse et al. [40]	Gearbox and Aero-Propulsion	Data-based	System health monitoring to detect and diagnose system problems and predict maintenance.
Bhatti et al. [41]	Industrial Robots	Hybrid	Detect and diagnose faults.
Modoni et al. [42]	Industry	Control Models	Improve the quality of produced micro manufactured devices.
Moghadam et al. [43]	Industry	Control Models	Monitor and estimate the fatigue of system components.
**Improve the Development Process**			
**Reference**	**Application**	**Modeling**	**Function**
Dong et al. [44]	Industry	Other	Propose product design improvements and innovations.
Fedorko et al. [45]	Industry	Control Models	Test physical properties in conveyor belts.
Li et al. [46]	Industry	Knowledge-based	Create more sustainable manufacturing methods to control environmental and social impacts.
Liu et al. [47]	Industry	Bayesian Network	Improve traceability and quality control in manufacturing processes.
Sun et al. [48]	Industry	Structural	Improve quality control and assembly efficiency in high-precision products.
**Training**			
**Reference**	**Application**	**Modeling**	**Function**
Cortes et al. [49]	Industry	Control Models	Teach industrial concepts and techniques to create qualified workforces.
Waat et al. [50]	Industry	Structural	Factory assembly training with AR technologies for new operators.

**Table 2:** Sample modeling techniques.

Behavior Model		
**Model Type**	**Characteristics**	**Examples**
Control Models	Based on control theory. They use the laws of physics and compare simulated results with known information, represented by mathematical models.	An et al. [25], Bottani et al. [26], Gonzalez et al. [28], Saad et al. [31], Schellenberger et al. [33], Xu et al. [35], Bartos et al. [39], Modoni et al. [42], Moghadam et al. [43], Fedorko et al. [45], Cortes et al. [49]
Data-Dependent Models	Based on artificial intelligence. They use data structures that retain all the variables describing the reality at a level of abstraction.	Stan et al. [22], Wang et al. [23], Huang et al. [30], Salvi et al. [32], Sousa et al. [34], Xu et al. [36], Barbi et al. [38], Booyse et al. [40]
Hybrid Control–Data Models	Combine control and data-dependent models to obtain the advantages of both of them.	Angjeliu et al. [37], Bhatti et al. [41]
Other Models	They use the relation of the components, e.g., graph, communication, process, ontology or knowledge-based models.	Bhatti et al. [24], Cainelli et al. [29], Dai et al. [68], Pylianidis et al. [69], Dong et al. [44], Li et al. [46], Liu et al. [47]
**Structural Model**		
**Model Type**	**Characteristics**	**Examples**
Physical Model	Represents physical properties and phenomena, such as deformation, cracking and corrosion.	Post et al. [70], Mathupriya et al. [10]
Geometrical Model	Reflects the geometry, shapes, sizes, positions, assembling machine components, kinematics, logic and interfaces of the real system.	Guo et al. [27], Sun et al. [48], Waat et al. [50]

**Table 3:** Sample model integration techniques.

Model Integration Technique		
**Integration Method**	**Characteristics**	**Examples**
Hierarchical	It builds complex systems by integrating smaller and simpler components.	Tao et al. [90], Borth et al. [91]
Collaborative	The different components interact and simulate a collaborative behavior among several assets.	Autiosalo et al. [92], Cimino et al. [93], Eramo et al. [94], Zheng et al. [95]

## Data Availability

Not applicable.
